# The Two-Stage Ipsilateral Fibular Transfer for Tibial Defect Following Tumour Excision

**DOI:** 10.1155/S1357714X00000050

**Published:** 2000-03

**Authors:** Masahito Hatori, Khalid S. Ayoub, Robert J. Grimer, Simon R. Carter, Roger M. Tillman

**Affiliations:** The Royal Orthopaedic Hospital Oncology Service The Royal Orthopaedic Hospital Birmingham UK

## Abstract

*Method.* We performed a two-stage vascularized ipsilateral fibular graft transfer for segmental tibial defect following excision of malignant bone tumours.

*Results.* We report 10 patients who had this procedure with an average follow-up of 116 months.The graft was transposed medialy on its vascular pedicle by two-stage surgery. Full weight bearing was achieved in six patients at 8–43 months postoperatively, but every patient had a significant complication.

*Discussion.* The use of this method in isolation is not recommended for reconstruction of the tibia following tumour excision.


Sarcoma (2000) 4, 27–30

ORIGINAL ARTICLE

### The two-stage ipsilateral fibular transfer for tibial defect following tumour excision

MASAHITO HATORI, KHALID S. AYOUB, ROBERT J. GRIMER, SIMON R. CARTER & ROGER M. TILLMAN

*The Royal Orthopaedic Hospital Oncology Service, The Royal Orthopaedic Hospital, Birmingham, UK*

**Abstract**

*Method.* We performed a two-stage vascularized ipsilateral fibular graft transfer for segmental tibial defect following excision of malignant bone tumours.

*Results.* We report 10 patients who had this procedure with an average follow-up of 116 months. The graft was transposed medialy on its vascular pedicle by two-stage surgery. Full weight bearing was achieved in six patients at 8–43 months postoperatively, but every patient had a significant complication.

*Discussion.* The use of this method in isolation is not recommended for reconstruction of the tibia following tumour excision.

**Key words:** *fibulograft, sarcoma, bone graft*

### Introduction

One of the most difficult problems confronting the orthopaedic surgeon is the reconstruction of large bone defects following resection of tumours, particularly arising in the tibial shaft. One of the common ways of reconstruction is autogenous cortical bone graft.[1–3] The majority of osteocytes of this graft undergo necrosis requiring the eventual replacement of the dead bone by creeping substitution.[4] Other methods of reconstruction including autoclaved bone[5] and allograft[6] have been used but frequently result in complications such as non-union and/or infection.[7,8] With the development of microvascular technique, free vascularized fibular grafts have been contributed followed by ipsilateral vascularized transfer.[4,9–11] However, such kinds of reconstructive procedures are extensive and time-consuming, requiring expensive equipment.[3] We report our experience of using two-stage ipsilateral fibular graft transfer as a method of tibial reconstruction following wide excision of malignant tumours arising in the shaft.

### Patients and methods

We have undertaken a retrospective review. Ten patients have undergone two-stage ipsilateral vascularized fibular transfer to reconstruct defects of the tibia. There are six males and four females. The ages range from 5 to 22 years with an average of 14 years. The mean follow-up is 116 months (range 19–175 months). The primary tumours of the affected tibia were: three osteosarcomas, three Ewing's sarcomas, two adamantinomas, one malignant fibrous histiocytoma, and one chondrosarcoma. The tumour locations in the tibial diaphysis are: one proximal tibial, six mid-tibial, and three distal tibial diaphysis. Pre- and post-operative chemotherapy was used for osteosarcoma and Ewing's sarcoma. The length of bone defect ranged from 8 to 20 cm with the average of 15 cm (Table 1).

### Operative technique

### *The first stage procedure*

Curved anterior incision was made, taking an ellipse of skin with the biopsy site. Dissection was continued medialy and laterally with care taken not to expose the tumour. Proximal and distal tibial osteotomies were performed excising the preoperatively-decided length of the tibia. The fibula was transected 1 cm beyond the level of distal tibial resection, mobilized and inserted into the medullary canal of the tibia (Fig. 1, left). If necessary, Kirschner wires or Steinmann pins were used for the stabilization of the fibula. Any remaining gap between the tibia and the fibula was filled with cancellous bone harvested from the

Correspondence to: Mr R.J. Grimer, Consultant Orthopaedic Oncologist, Royal Orthopaedic Hospital Oncology Service, The Royal Orthopaedic Hospital NHS Trust, Bristol Road South, Northfield, Birmingham B31 2AP, UK. Tel.: 0121-6854150; Fax: 0121-6854146.

1357-714Xprint/1369-1643online/00/010027-04 © 2000 Taylor & Francis Ltd

**Table 1**. *Details of 10 patients with fibular graft*

| Patient | Gender, age (years) | Length of tibial defect (cm) | Complications | Treatment | Time to union (months) | Time to full weight bearing (months) | Follow-up (months) | Status at latest follow-up |
|---|---|---|---|---|---|---|---|---|
| LH | F, 5 | 10 | Local recurrence | Below-knee amputation | | | 19 | Died of lung metastasis |
| LW | M, 6 | 10 | Stress fracture | Non-operative | 2 | 19 | 137 | No evidence of disease |
| SI | M, 8 | 16 | Non-union, valgus deformity, mild pin infection | Two operations of bone grafting | 21 | 24 | 108 | No evidence of disease |
| PL | M, 13 | 15 | 2.5 cm shortening | | Not available | Not available | 169 | No evidence of disease |
| MN | M, 15 | 14 | Varus deformity | | 3 | 8 | 116 | No evidence of disease |
| JW | F, 17 | 20 | Stress fracture, equinus, 2 cm shortening | Non-operative treatment for stress fracture | 12 | 20 | 108 | No evidence of disease |
| MB | F, 18 | 18 | Non-union, stress fracture | Two operations of bone grafting | 24 | 33 | 149 | No evidence of disease |
| PS | M, 19 | 18 | Residual tumour, lung metastasis | Below-knee amputation | | | 89 | No evidence of disease |
| MG | F, 20 | 8 | (a) Traumatic fracture (b) Stress fracture | (a) Ex. fixation + bone grafting (b) Non-operative | 6 | 43 | 90 | No evidence of disease |
| DW | M, 22 | 13 | (a) Non union (b) Stress fracture (c) Local recurrence | (a) Three operation of bone grafting (b) Non-operative (c) Below-knee amputation | 32 | Never occurred | 175 | Died of lung metastasis |

osteotomized tibial edges. The wound was closed in layers. The operating time was less than 90 min. Post-operative casting was applied.

*The second stage procedure*

The second stage surgery was performed at 2–4 months, with the average of 3 months, after the first procedure. The fibula was divided 1–2 cm above the level of the tibia (Fig. 1, right). A window was created in the cortex of the tibia and the fibula was slotted in and packed with bone graft taken from the proximal tibial segment. Union was assessed clinically and radiographicaly at regular intervals. If there was evidence of non-union, the site was surgically exposed and plated with autogenous cancellous bones. Stress fractures were treated by plaster casting.

### Results

Out of 10 patients included in this study, wide surgical excision of the tumour was achieved in nine. One patient had an intra-lesional excision of the primary tumour at the first stage procedure and was treated with a below-knee amputation. Local recurrence occurred in two patients, both treated with below-knee amputations. Metastasis developed in three patients, two of them died as a result. The remaining eight patients are still alive and free of disease.

Bone union between the fibular graft and the tibia occurred in five patients with an average time to union of 6 months (range 2–12 months). Three patients developed non-union. These were treated by internal fixation (plating) and secondary bone grafting resulting to full union and incorporation of the graft with the tibia. The other two patients had early below-knee amputations (one local recurrence and one residual tumour) before reaching the stage of complete union. Five fractures (four stress fractures and one traumatic) of the fibular strut were diagnosed in four patients at 10, 13, 13, 19 and 24 months after the second stage procedure. These developed during the rehabilitation period with increasing weight bearing on the operated leg. The stress fractures were treated by conservative (non-operative) methods, and the traumatic fracture by external fixation with bone grafting. All resulted in complete union and gave good results. Full and unprotected (i.e. no splintage) weight-bearing on the operated leg, with or without walking aids, was achieved at an average time of 21 months (range 8–43 months). We found that the main causes for this rather prolonged period until full weight bearing were stress fractures and non-union.

Radiographically, hypertrophy of the grafted fibula was observed in all the patients (Fig. 2).

The range of knee and ankle movements were

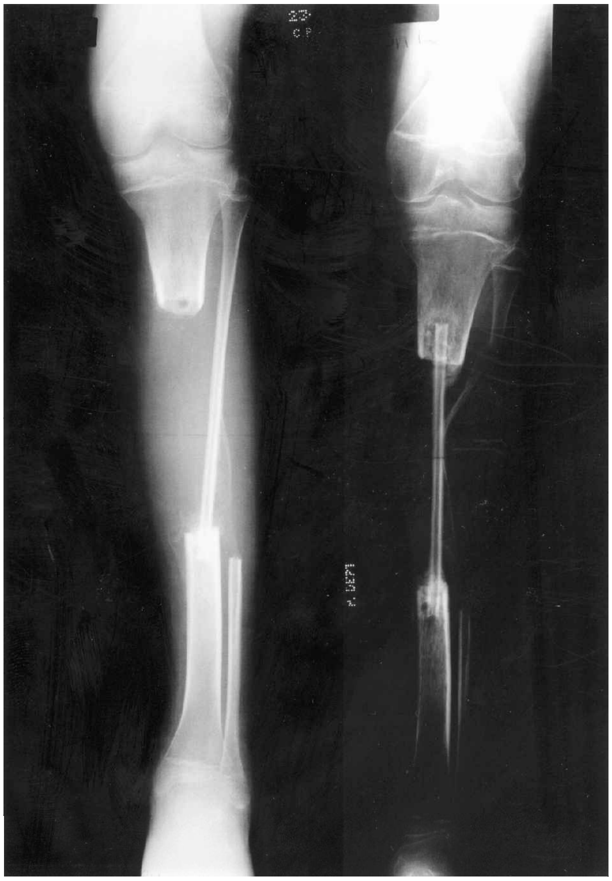

**Fig. 1.** *Anteroposterior view of postoperative radiographs of 15-year-old boy who had a malignant fibrous histiocytoma in the mid-shaft left tibia.* Left: *Two months after the first stage procedure showing 14 cm tibial bone defect following tumour resection, and the distal fibular osteotomy and transfer into the canal of distal tibial segment.* Right: *One month after the second stage procedure showing the proximal fibular osteotomy and transfer into the canal of proximal tibial segment.*

normal in seven and five patients, respectively. In two patients the range of ankle motion was impaired due to mild equinus deformity. The axial alignment was normal in five patients. Asymptomatic valgus and varus deformities were observed in one patient each. Two centimetres of leg shortening was found in two patients. Mild pin infection was observed in one patient. No other local or systemic complications have occurred (Table 1).

### Discussion

Wide excision of a lesion in the tibia presents a difficult reconstructive problem in that it produces an intercalary gap in a major weight-bearing bone. The main goal is restoration of the skeletal anatomy and function of the extremity. With the development of new technology, the surgeon has a wide selection of available methods with which to bridge the massive bone defects that are created by the radical resection of large tumours of bone.[3]

The fibula was used as a substitute for a missing segment of tibia or to reinforce a weakened section using different methods. Transplantation of the upper end of the fibula into the tibia to replace loss of tibial substance was first conceived by Hahn in 1884.[12] Huntington took the procedure one stage further and transferred the lower end of the fibula into the tibia.[13] Wilson in 1941 published his experience in two-stage fibular transplantation for use in cases of complicated and congenital pseudoarthrosis of the tibia. He stressed that the operation is best performed in two stages because this method has the advantages of lessening the risk of dislodging the end of the fibula from its connection with the tibia, of shortening the time of the operation, and of reducing the severity of the postoperative reaction.[14]

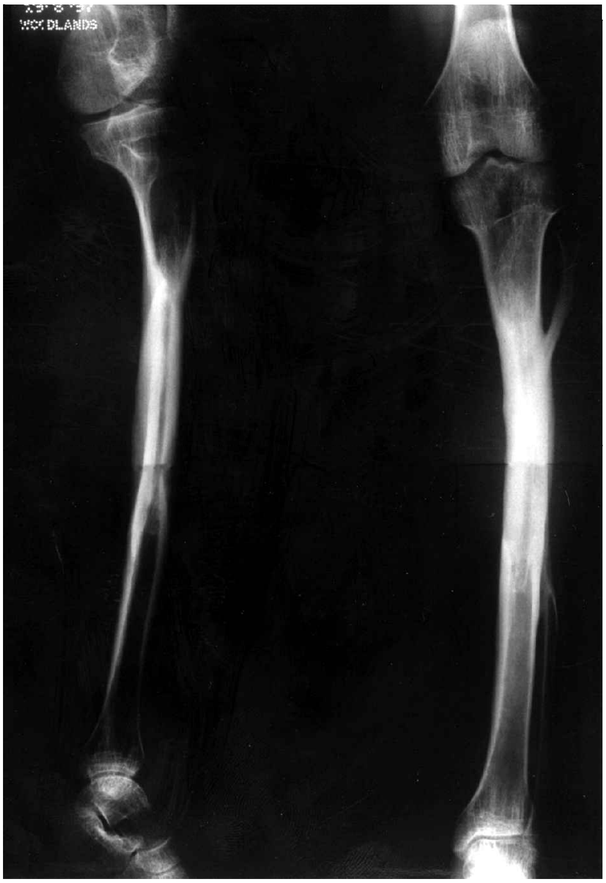

**Fig. 2.** *Anteroposterior and lateral radiographs for the same patient in Fig. 1, almost 8 years following the second stage procedure. It shows complete incorporation of the graft with the tibia, as well as hypertrophy of the graft itself acquiring the size and shape of the tibia.*

The shaft of the fibula is strong and could provide a free vascularized graft from the contralateral side using a microvascular technique (Taylor, Miller and Ham). Many authors have reported the usefulness of the free vascularized fibula graft.[9,15] However, this technique is highly specialized and may not be available even in major centres.[16] A high complication rate has been reported.[17] It also means operating on the non-affected leg and sacrificing the contralateral fibula, and that should be performed before tumour excision in order to minimize the possibility of tumour cells seeding.

The development of techniques for harvesting microvascular fibular transplants indicated the potential use of the ipsilateral fibula, retaining the periosteal and endosteal circulation.[10,16] Chacha *et al.* carried out the dissection and transposition of the fibula, preserving its vascular supply in 11 patients. He was successful in obtaining good results. The use of the ipsilateral fibula with an intact blood supply through its musculoperiosteal vessels provides a large living graft which can be fixed to the tibia. The experimental and the clinical studies have provided good evidence of the viability of such a graft.[16] Other authors also reported the usefulness of this method for the treatment of massive bone defect of the tibia, mainly due to trauma.[10,11,18] Although this procedure has many merits, it is thought to be time-consuming, technically demanding and one-stage fibula transfer is not always possible in terms of its vascular preservation. Other options for reconstructing the tibia need to be considered. These include the option of bone transport, allograft, and the combined option of ipsilateral vascularized fibular graft with allograft.[19]

On the basis of our experience we do not recommend an ipsilateral fibular graft as a good option for limb reconstruction in isolation in the tibia. In our experience no patient avoided a significant complication, and also underwent the uncertainty of a prolonged period of partial or non-weight bearing with consequent diminished function.

Having reviewed the literature on this subject we feel that the most encouraging results have been with a combination of vascularized fibular graft with an allograft, and we have currently used this in several patients with promising results. For short segment bone resection we have used bone transport. In general there is no 'best option' and the technique used will depend on the availability of allograft, the availability of appropriate external fixator skills and the possibility of micro-vascular reconstruction.

### References

1 Enneking WF, Eady JL, Burchardt H. Autogenous cortical bone grafts in the reconstruction of segmental skeletal defects. *J Bone Joint Surg* 1980; 62A:1039–58.
2 Swierstra BA, Rijnberg WJ, van Linge B. Reconstruction of the tibia by dual grafting. 3 cases of tumour resection. *Acta Orthop Scand 1990*; 61:266–8.
3 Yadav SS. Dual-fibular grafting for massive bone gaps in the lower extremity. *J Bone Joint Surg* 1990; 72A:486–94.
4 Takami H, Takahashi S, Ando M, Masuda A. Vascularized fibular grafts for the resonstruction of segmental tibial bone defects. *Acta Orthop Trauma Surg* 1997; 116:404–7.
5 Sijbrandij S. Resection and reconstruction for bone tumours. *Acta Orthop Scand* 1978; 49:249–54.
6 Gebhardt MC, Lord FC, Rosenberg AE, Mankin HJ. The treatment of adamantinoma of the tibia by wide resection and allograft bone transplantation. *J Bone Joint Surg* 1987; 69A:1177–88.
7 Mankin HJ, Doppelt S, Tomford W. Clinical experience with allograft implantation. The first ten years. *Clin Orthop* 1983; 174:69–86.
8 Ortiz-Cruz E, Gebhardt MC, Jennings LC, Springfield DS, Mankin HJ. The results of transplantation of intercalary allografts after resection of tumors. *J Bone Joint Surg* 1997; 79A:97–106.
9 Taylor GI, Miller GD, Ham FJ. The free vascularized bone graft: a clinical extension of microvascular techniques. *Plast Recontr Surg* 1975; 55:533–44.
10 Hertel R, Pisan M, Jakob RP. Use of the ipsilateral vascularized fibula for tibial reconstruction. *J Bone Joint Surg (Br)* 1955;77B:914–19.
11 Shapiro MS, Endrizzi DP, Cannon RM, Dick HM. Treatment of tibial defects and nonunion using ipsilateral vascularized fibular transposition. *Clin Orthop* 1993; 296:207–12.
12 Hahn Eugen. Eine Methode, Pseudarthrosen der Tibia mit grossem Knochendefekt zur Heilung zu bingen. *Zentralbl f Chir* 1884; 11:337–41
13 Huntington TW. Case of bone transference. Use of a segment of fibula to supply a defect in the tibia. *Ann Surg* 1905; 41:249–51.
14 Wilson PD. A simple method of two-stage transplantation of the fibula for use in cases of complicated and congenital pseudoarthrosis of the tibia. *J Bone Joint Surg* 1941; 23:639–75.
15 Weiland AJ, Daniel RK. Microvascular anastomoses for bone grafts in the treatment of massive defects in bone. *J Bone Joint Surg* 1989; 61A:98–104.
16 Chacha PB, Ahmed M, Daruwalla JS. Vascular pedicle graft of the ipsilateral fibula for non–union of the tibia with a large defect: an experimental and clinical study. *J Bone Joint Surg (Br)* 1981; 63B:244–53.
17 Hsu RW-W, Wood MB, Sim FH, Chao ES. Free vascularized fibular grafting for reconstruction after tumour resection. *J Bone Joint Surg* 1997; 79B:36–42.
18 Coleman SS, Coleman DA. Congenital pseudoarthrosis of the tibia: treatment by transfer of the ipsilateral fibula with vascular pedicle. *J Pediatr Orthop* 1994; 14:156–60.
19 Ozaki A, Hillmann A, Wuisman P, Winkelmann W. Reconstruction of tibia by ipsilateral vascularized fibula and allograft. 12 cases with malignant bone tumors. *Acta Orthop Scand (Norway)* 1997;68:298–301.

## References

[PDF_00375] Chacha P. B., Ahmed M., Daruwalla J. S. (1981). Vascular pedicle graft of the ipsilateral fibula for non-union of the tibia with a large defect. An experimental and clinical study.. J Bone Joint Surg Br.

[PDF_00382] Coleman S. S., Coleman D. A. (1994). Congenital pseudarthrosis of the tibia: treatment by transfer of the ipsilateral fibula with vascular pedicle.. J Pediatr Orthop.

[PDF_00326] Enneking W. F., Eady J. L., Burchardt H. (1980). Autogenous cortical bone grafts in the reconstruction of segmental skeletal defects.. J Bone Joint Surg Am.

[PDF_00341] Gebhardt M. C., Lord F. C., Rosenberg A. E., Mankin H. J. (1987). The treatment of adamantinoma of the tibia by wide resection and allograft bone transplantation.. J Bone Joint Surg Am.

[PDF_00379] Hsu R. W., Wood M. B., Sim F. H., Chao E. Y. (1997). Free vascularised fibular grafting for reconstruction after tumour resection.. J Bone Joint Surg Br.

[PDF_00365] Huntington T. W. (1905). VI. Case of Bone Transference: Use of a Segment of Fibula to Supply a Defect in the Tibia.. Ann Surg.

[PDF_00345] Mankin H. J., Doppelt S., Tomford W. (1983). Clinical experience with allograft implantation. The first ten years.. Clin Orthop Relat Res.

[PDF_00348] Ortiz-Cruz E., Gebhardt M. C., Jennings L. C., Springfield D. S., Mankin H. J. (1997). The results of transplantation of intercalary allografts after resection of tumors. A long-term follow-up study.. J Bone Joint Surg Am.

[PDF_00386] Ozaki T., Hillmann A., Wuisman P., Winkelmann W. (1997). Reconstruction of tibia by ipsilateral vascularized fibula and allograft. 12 cases with malignant bone tumors.. Acta Orthop Scand.

[PDF_00358] Shapiro M. S., Endrizzi D. P., Cannon R. M., Dick H. M. (1993). Treatment of tibial defects and nonunions using ipsilateral vascularized fibular transposition.. Clin Orthop Relat Res.

[PDF_00339] Sijbrandij S. (1978). Resection and reconstruction for bone tumours.. Acta Orthop Scand.

[PDF_00329] Swierstra B. A., Rijnberg W. J., van Linge B. (1990). Reconstruction of the tibia by dual grafting. 3 cases of tumor resection.. Acta Orthop Scand.

[PDF_00335] Takami H., Takahashi S., Ando M., Masuda A. (1997). Vascularized fibular grafts for the reconstruction of segmental tibial bone defects.. Arch Orthop Trauma Surg.

[PDF_00352] Taylor G. I., Miller G. D., Ham F. J. (1975). The free vascularized bone graft. A clinical extension of microvascular techniques.. Plast Reconstr Surg.

[PDF_00332] Yadav S. S. (1990). Dual-fibular grafting for massive bone gaps in the lower extremity.. J Bone Joint Surg Am.

